# Preparation and Cyclodextrin Solubilization of the Antibacterial Agent Benzoyl Metronidazole

**DOI:** 10.1155/2013/306476

**Published:** 2013-07-21

**Authors:** Qing Huang, Benpeng Li, Shuo Yang, Peipei Ma, Zhizhong Wang

**Affiliations:** School of Pharmacy, Ningxia Medical University, Yinchuan, Ningxia 750004, China

## Abstract

A one-pot method for the preparation of benzoyl metronidazole was achieved by using *N,N*′-carbonyldiimidazole as a coupling reagent. Moreover, it was found that the byproduct imidazole as the catalyst promoted the reaction. In addition, the **β**-cyclodextrin solubilization of benzoyl metronidazole was investigated by phase-solubility method. The phase-solubility studies indicated that the solubility of benzoyl metronidazole (*S* = 0.1435 g/L) was substantially increased 9.7-fold (*S*′ = 1.3881 g/L) by formation of 1 : 1 benzoyl metronidazole/**β**-cyclodextrin complexes in water, and the association constant *K*_*a*_ value was determined to be 251 (±23) dm^3^/mol. Therefore, **β**-cyclodextrin can work as a pharmaceutical solubilizer for benzoyl metronidazole and may improve its oral bioavailability.

## 1. Introduction

Metronidazole (MTZ), 2-(2-methyl-5-nitro-1*H*-imidazol-1-yl)ethanol, is known to be a powerful antiprotozoal and antibacterial drug. It is clinically effective in trichomoniasis, amoebic colitis, and giardiasis [1]. However, it has a bitter taste and is not acceptable to some young patients. Benzoyl metronidazole, the benzoyl ester of metronidazole, is tasteless and has also been widely used because of its greater palatability [2]. In addition, formulation of benzoyl metronidazole in aqueous drug formulation has been hampered by its low aqueous solubility.

**Scheme 1:**
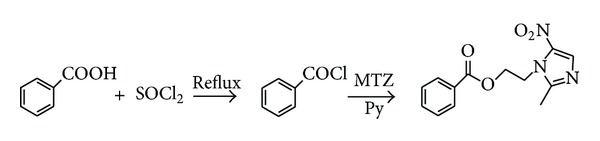


**Scheme 2:**
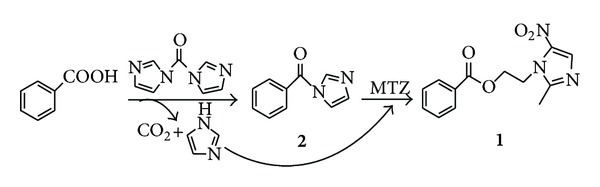


The conventional routes for synthesis of benzoyl metronidazole were the two-step synthesis as shown in Scheme 1, which required the preparation of benzoyl chloride; the reaction required strict anhydrous conditions [3, 4], and the reactor should be corrosion resistant. In addition, in the combination of benzoyl chloride and metronidazole, the deacid reagent such as pyridine was needed to promote the reaction. *N,N*′-carbonyldiimidazole (CDI) is one of several commonly used reagents for activating carboxyl groups. It is relatively cheap, and the only byproducts are carbon dioxide and imidazole which, being relatively benign, are unlikely to cause problems on scale up [5]. Herein, we report an improved procedure for the preparation of benzoyl metronidazole in one pot by using *N,N*′-carbonyldiimidazole as a coupling reagent, and the byproduct imidazole as the catalyst can promote the reaction (Scheme 2). In addition, the use of **β**-cyclodextrin (**β**-CD) to increase the water solubility of benzoyl metronidazole was described.

## 2. Materials and Methods

### 2.1. Materials and Instruments

All the experiments were performed with analytical-grade chemicals and solvents. **β**-CD was recrystallized twice from distilled water and dried under reduced pressure at 110°C for 24 h before use. Dichloromethane (DCM) was dried by CaCl_2_ for 12 h and distilled prior to use. The progresses of the reactions were monitored by TLC on 0.25 mM thick layers of silica gel GF_254_ developed with solvent system, AcOEt : petroleum ether (1 : 1, v/v). ^1^H NMR spectra were recorded on Bruker 400 spectrometer in CDCl_3_ solutions with TMS (tetramethylsilane) as standard. The melting point determinations were carried out on a XRC-I melting point apparatus. The spectroscopic measurements were performed on a double-beam UV-vis spectrophotometer (Shimadzu UV-2550).

### 2.2. Facile Preparation of Benzoyl Metronidazole

To a solution of benzoic acid (1.0 g, 8.2 mmol) in 40 cm^3^ anhydrous DCM, *N,N*′-carbonyldiimidazole (1.6 g, 9.8 mmol) was added at room temperature. After being stirred for 4 h, metronidazole (1.4 g, 8.2 mmol) in 100 cm^3^ anhydrous DCM was added. Then the resulting solution was refluxed for 10 h and concentrated. The residue was dissolved in 80 cm^3^ DCM and sequently washed with 1 M sodium carbonate solution (2 × 20 cm^3^), 10% HCl (2 × 15 cm^3^), and distilled water (3 × 20 cm^3^). The organic layer was separated, dried over anhydrous sodium sulphate, and concentrated in vacuo. Finally, the concentrate was crystallized from ethanol to give **1** (1.97 g). Yield 87%; M.p.: 99.3–100.4°C ([6] 99–102°C). ^1^H NMR (400 MHz, CDCl_3_): *δ* 2.49 (s, 3H, CH_3_ group, imidazole), 4.69 (t, 2H, N–CH_2_), 4.72 (t, 2H, O–CH_2_), 7.42–7.93 (m, 5H, Ar–H), and 7.98 (s, 1H, imidazole ring H).

### 2.3. Solubility Measurements

Phase-solubility studies of benzoyl metronidazole in aqueous solutions of **β**-CD were carried out according to the Higuchi-Connors procedure [7]. Various amounts of **β**-CD were generally dissolved in distilled water, and excess amounts of benzoyl metronidazole were loaded in glass vials. The vials were shaken in a temperature-controlled room at 298 K for 24 h to achieve the equilibrium. Appropriate aliquots were then withdrawn and filtered appropriately diluted with distilled water, and the total concentration of benzoyl metronidazole in the filtrate was analyzed by UV-vis absorbance spectrum. The absorbances at 316 nm for benzoyl metronidazole were measured, in order to determine the concentration of the dissolved benzoyl metronidazole.

## 3. Results and Discussion

### 3.1. Facile Preparation of Benzoyl Metronidazole

In our work, we found that simply mixing benzoyl imidazole **(2)** with metronidazole in DCM did not result in an obvious reaction in 24 h. Imidazole can efficiently promote the reaction. Furthermore, the stronger bases such as Na_2_CO_3_ or Et_3_N did not distinctly enhance the reaction rate.

*N,N*′-carbonyldiimidazole is a useful, general carboxylic acid activating reagent, and its byproduct imidazole can serve as the catalyst. Therefore, benzoyl metronidazole **(1)** was prepared in a simple, two-step sequence in just one reactor. In the first step, based on TLC analysis, the reaction between benzoic acid and *N,N*′-carbonyldiimidazole was quantitative within 4 h at room temperature. In the second step, the coupling of metronidazole to the benzoyl group was accomplished in 10 h under reflux in DCM.

The optimized conditions for the synthesis of benzoyl metronidazole are benzoic acid/*N,N*′-carbonyldiimidazole/metronidazole = 1/1.2/1 (mol/mol). The purity and structure were confirmed by NMR, TLC, and melting point.

### 3.2. Solubilization of Benzoyl Metronidazole by *β*-CD

Benzoyl metronidazole possesses poor solubility in water (*S* = 0.1435 g/L, 5.22 × 10^−4^ M). Solubilization of poorly soluble drugs is one of the most important physicochemical properties for drug development since more than one-third of drugs are poorly water soluble or water insoluble [8]. In order to prepare a liquid dosage formulation of these drugs, a solubilization technique is usually applied. **β**-CD has been widely used as an excipient in the pharmaceutical industry for improving some properties of drugs, such as solubility, stability, absorption, and/or bioavailability, by forming the inclusion complexes [9]. In our work, the **β**-CD solubilization of benzoyl metronidazole in purified water was first assessed. The phase-solubility studies indicated that **β**-CD increased the aqueous solubility of benzoyl metronidazole by approximately 9.7-fold (*S*′ = 1.3881 g/L, 5.05 × 10^−3^ M).

**Figure 1:**
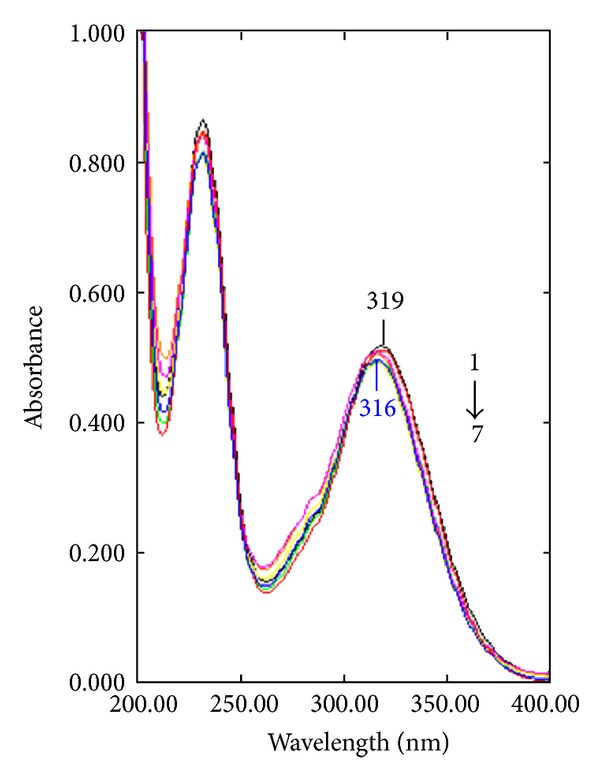
UV-vis absorption spectra of benzoyl metronidazole (5.7 × 10^−5^ M) at various concentrations of **β**-CD at 298 K: (1) no **β**-CD, (2) 1.6 mM, (3) 3.2 mM, (4) 4.8 mM, (5) 8.0 mM, (6) 11.2 mM, and (7) 14.4 mM **β**-CD.

**Figure 2:**
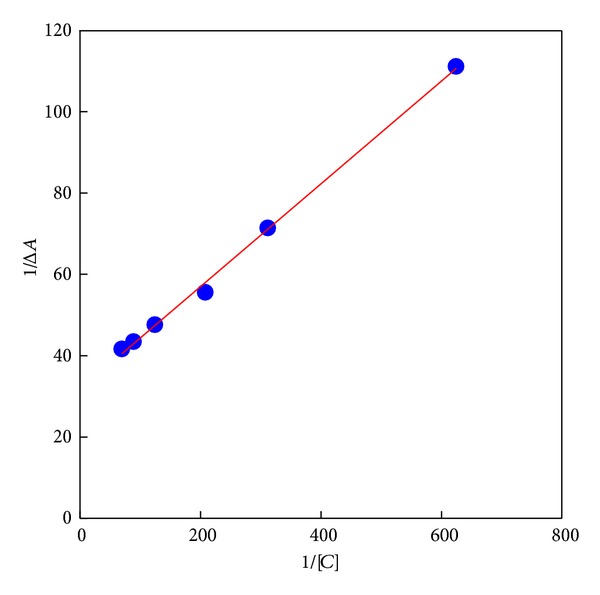
Benesi-Hildebrand plot for the complexation of benzoyl metronidazole with **β**-CD.

The decreasing of the UV-vis absorbance intensity and the blue shifted absorbance maximum suggest the formation of an inclusion complex between benzoyl metronidazole and **β**-CD (Figure 1). The linearity in the plot revealed the formation of 1 : 1 complex between benzoyl metronidazole and **β**-CD (Figure 2). The stability constant *K*_*a*_ can be determined by using Benesi-Hildebrand equation [10]:

(1) $$\begin{array}{c} \frac{1}{\Delta A}=\frac{1}{\Delta \varepsilon \left[S\right]}+\frac{1}{\Delta \varepsilon K_{a}\left[S\right]\left[C\right]}, \end{array}$$

where [*S*] and [*C*] represent the concentrations (mol/dm^3^) of substrate and **β**-CD, respectively. Δ*A*  is the change in the absorbance of the substrates before and after addition of **β**-CD, and Δ*ε* is the difference in the molar absorptivities between complexed and free substrate. Plotting 1/Δ*A* against 1/[*C*] gives a straight line with slope equal to 1/Δ*εK*_*a*_[*S*]. The association constant *K*_*a*_ was directly obtained from the intercept/slope ratio. The *K*_*a*_ value was determined to be 251(±23) dm^3^/mol for the inclusion complexation of **β**-CD with benzoyl metronidazole.

## 4. Conclusions

An improved method for the preparation of benzoyl metronidazole via a one-pot reaction was developed by using *N,N*′-carbonyldiimidazole as a coupling reagent, which does not require extra catalysts. Compared with routine synthetic methods, these procedures may become an efficient route for the synthesis of benzoyl metronidazole. In addition, the phase-solubility studies indicated that the solubility of benzoyl metronidazole (*S* = 0.1435 g/L) was substantially increased 9.7-fold (*S*′ = 1.3881 g/L) by formation of 1 : 1 benzoyl metronidazole/**β**-CD complexes in water, and the association constant *K*_*a*_ value was determined to be 251(±23) dm^3^/mol. Therefore, **β**-CD can work as a pharmaceutical solubilizer for benzoyl metronidazole and may improve its oral bioavailability.
